# Trait matching in a multi‐species geographic mosaic of leafflower plants, brood pollinators, and cheaters

**DOI:** 10.1002/ece3.10228

**Published:** 2023-07-04

**Authors:** Kai Hao, Ting‐Ting Liu, David H. Hembry, Shi‐Xiao Luo

**Affiliations:** ^1^ Key Laboratory of Plant Resources Conservation and Sustainable Utilization, South China Botanical Garden Chinese Academy of Science Guangzhou China; ^2^ South China National Botanical Garden Guangzhou China; ^3^ Department of Biology University of Texas Permian Basin Odessa Texas USA

**Keywords:** brood pollination mutualism, cheater leafflower moth, coadaptation, geographic mosaic of coevolution, multi‐species assemblages, trait matching

## Abstract

Trait matching between mutualistic species is usually expected to maintain mutualism, but empirical studies of trait complementarity and coadaptation in multi‐species assemblages—which represent the reality of most interactions in nature—are few. Here, we studied trait matching between the leafflower shrub *Kirganelia microcarpa* and three associated seed‐predatory leafflower moths (*Epicephala* spp.) across 16 populations. Behavioral and morphological observations suggested that two moths (*E. microcarpa* and *E. tertiaria*) acted as pollinators while a third (*E. laeviclada*) acted as a cheater. These species differed in ovipositor morphology but showed trait complementarity between ovipositor length and floral traits at both species level and population level, presumably as adaptations to divergent oviposition behaviors. However, this trait matching varied among populations. Comparisons of ovipositor length and floral traits among populations with different moth assemblages suggested an increase of ovary wall thickness where the locular‐ovipositing pollinator *E. microcarpa* and cheater *E. laeviclada* were present, while stylar pit depth was less in populations with the stylar pit‐ovipositing pollinator *E. tertiaria*. Our study indicates that trait matching between interacting partners occurs even in extremely specialized multi‐species mutualisms, and that although these responses vary, sometimes non‐intuitively, in response to different partner species. It seems that the moths can track changes in host plant tissue depth for oviposition.

## Graphical abstract

Moth ovipositor morphology matches with different floral traits, suggesting potential adaptation to divergent oviposition behaviors across populations. Floral traits vary similarly in response to locular oviposition by both one of the pollinator species and the cheater, suggesting the possibility of arms race dynamics between plants and insect pollinators or cheaters.

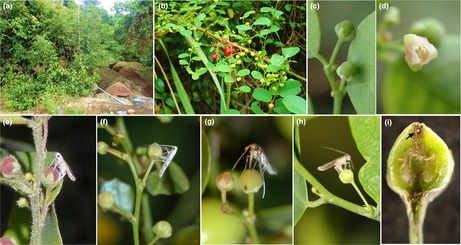

## 1 INTRODUCTION

Trait matching between partners is usually expected to maintain mutualistic interactions, since in order for mutualisms to function, partner taxa must have traits which match, and thus such matching traits will be under strong stabilizing selection (Week & Nuismer, 2021; Yoder & Nuismer, 2010). In its most extreme forms, such as yuccas and yucca moths, and figs and fig wasps, insects and their host plants exhibit species‐specific reciprocal adaptation in both morphology and behaviors pertaining to pollination and oviposition (Althoff & Segraves, 2022; Kjellberg et al., 2001). At the same time, other mutualisms show coevolutionary arms race dynamics, in which trait mismatching is favored on one side of the interaction and trait matching is favored on the other (Anderson et al., 2010; Darwin, 1862; Nilsson, 1988). Brood pollination mutualisms may contain elements of both such dynamics because they contain both mutualistic (pollination) and antagonistic (seed predation) components (Althoff & Segraves, 2022). Despite the importance of trait matching and mismatching in mutualistic interactions, they have been primarily empirically demonstrated in specialized associations with few interacting species. However, interactions in nature are usually not one‐to‐one, but involve multiple interacting partners in multi‐species assemblages (Hollens‐Kuhr et al., 2021; Thompson, 2005). Consequently, testing trait matching between multiple partners is of great importance to understand the evolution and maintenance of mutualisms.

Not only are most mutualisms embedded in multi‐species networks of mutualistic species, empirical, and theoretical studies indicate that antagonists may be common or at least important in many mutualistic systems (Genini et al., 2010; Jones et al., 2015). Third‐party exploiters or “cheaters” have been demonstrated in many mutualisms, including non‐pollinating parasites which evolved from pollinating ancestors (cheater yucca moths and cheater fig wasps; Machado et al., 2001; Pellmyr et al., 1996; Zhang et al., 2021), non‐cooperative symbionts in plant‐rhizobia interactions (Gano‐Cohen et al., 2020), and nectar‐robbing from flowers by bees and birds (Irwin & Brody, 1998; Rojas‐Nossa et al., 2016). Numerous studies have proposed explanations for why antagonists persist in mutualistic assemblages (Ferriere et al., 2002; Sachs & Simms, 2006), including the important observation that mutualists and antagonists may coexist in varied local assemblages in a geographic mosaic of different species at different sites (Hollens‐Kuhr et al., 2021; Thompson, 2005). However, empirical studies describing the spatial pattern of interactions among multiple mutualists and cheaters, and corresponding trait matching, are lacking.

Although there is ample evidence that mutualism promotes stabilizing selection, mutualism may also influence population phenotypic differentiation and speciation depending on specificity, partner dependence and environmental context (Althoff, 2014; Zeng & Wiens, 2020). In obligate mutualisms, partners are highly specialized and functional traits are reciprocally adapted to each other, so phenotypic differentiation presumably will greatly affect trait matching between partners leading to host shifts, partner replacement, and rapid reproductive isolation. For most insects, ovipositor (egg‐laying structure) morphology is critical for both host plant use (Joy & Crespi, 2007) and copulation (Althoff, 2014), which is tied directly to reproductive fitness and reproductive isolation. For example, the ovipositor morphology of *Tegeticula* moths shifts from long, thin ovipositors to short, thick ovipositors, along with the shift of oviposition habit from locular oviposition to superficial oviposition, which maintains the reproductive isolation among species using the same yucca host (Althoff, 2014; Althoff et al., 2006). Furthermore, phylogenetic analyses suggested that speciation of at least 11 species of yucca moths was coupled with changes in ovipositor morphology (Althoff et al., 2006; Darwell et al., 2016).

In this study, we examine trait matching in local assemblages of mutualists and cheaters using one of the best‐known specialized brood pollination mutualisms, leafflower–leafflower moth associations. Leafflower moths in the genus *Epicephala* (Gracillariidae) moths are specific to monoecious host plants in the family Phyllanthaceae (Kato et al., 2003; Luo et al., 2017) and have served as a model system in the study of plant–insect coevolution. Female moths visit male flowers to collect pollen grains with ciliated proboscises (Figure S1) and deposit pollen grains on the stigmas of female flowers by inserting their proboscises into the flower's narrow stylar pit. Female moths subsequently lay eggs in the pollinated flowers and their larvae consume some of the host's seeds. Similar to yucca–yucca moth and fig–fig wasp systems, interactions between leafflowers and leafflower moths are not absolutely one‐to‐one: It is common for multiple *Epicephala* pollinators to coexist in some Phyllanthaceae species, or even coexist with cheaters, which lay eggs in the flowers but provide no pollination service (Kawakita et al., 2015; Kawakita & Kato, 2006). The inability of cheaters to pollinate flowers is due to the loss of hairs on the surface of proboscises, which in pollinating species is used to collect and transport pollen grains (Kawakita & Kato, 2006).

Different *Epicephala* species differ in ovipositor morphology and oviposition behaviors. For example, the eggs are laid in the interspace between calyx lobes and carpel walls by moths associated with *Breynia vitis‐idaea*, *B. fruticosa*, *B. rostrata*, *Dendrophyllanthus bourgeoisii*, and *D. aeneus* (Kawakita & Kato, 2004a, 2004b; Zhang et al., 2012), laid at the basal part of ovary by laterally cutting through calyx lobes and ovary by other moths associated with *B. fruticosa* and *B. rostrata* (Kawakita & Kato, 2004b; Zhang et al., 2012), laid above ovules by inserting the ovipositor into the stylar pit by moths associated with *Glochidion acuminatum* and *G. coccineum* (Chheang et al., 2022; Kato et al., 2003), laid inside the ovaries by ovipositing through the carpel wall beside the stylar pit by *Epicephala lanceolaria* (Luo et al., 2017), and laid inside the ovaries by inserting the ovipositor through the base of the stylar column by moths associated with *G. temehaniense* (Hembry et al., 2012). In addition, Kawakita and Kato (2016) studied the morphology of six *Epicephala* moths and hypothesized an association between an angular ovipositor and a behavior of laterally cutting through the ovary wall. In the present study, we investigated the Asian leafflower shrub *Kirganelia microcarpa* and its three associated *Epicephala* moths in 16 populations across southern China. We aimed to answer the three following questions: (1) What is the spatial pattern of pollinator and cheater coexistence? (2) Do ovipositor morphology and corresponding oviposition behaviors match different floral traits? (3) Do the patterns of coexistence of pollinators and the cheater affect floral traits and trait matching between plant and pollinators?

## 2 MATERIALS AND METHODS

### 2.1 Studied species

**FIGURE 1:**
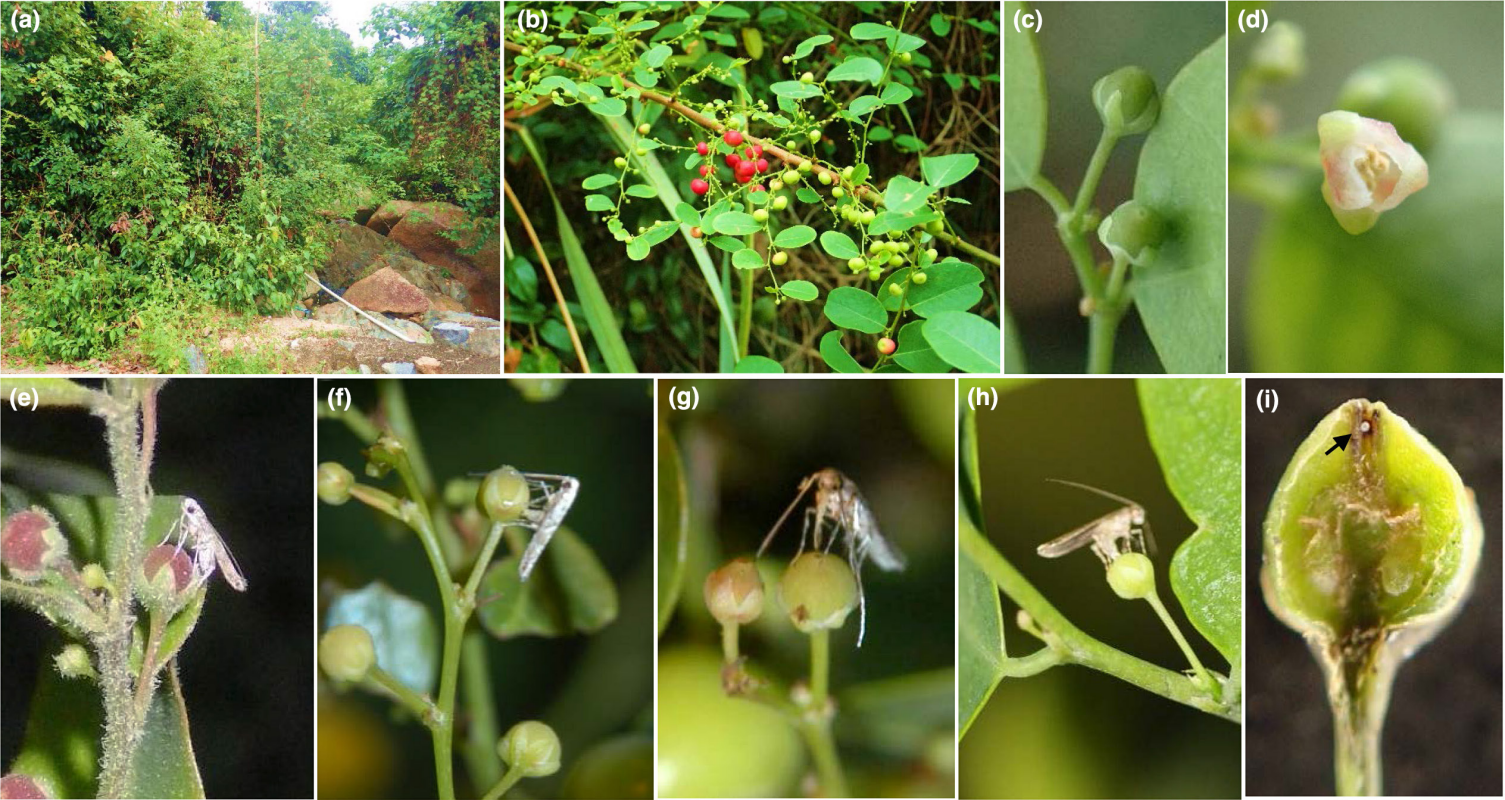
Habitat (a), inflorescences and immature fruits (b), female flower (c), and male flower (d) of *Kirganelia microcarpa*; pollination behavior of the moth *Epicephala microcarpa*, in which a female moth inserts her proboscis into the stylar pit (e), and oviposition behavior in which she laterally cuts through the ovary wall (f); pollination (g) and oviposition (h) behavior of the moth *E. tertiaria*, in which a female moth inserts her proboscis and then her ovipositor into the stylar pit; egg (i) and larva of *Epicephala* sp. in stylar pit of the flower (white arrows).

*Epicephala* moths (Lepidoptera: Gracillariidae) actively pollinate and oviposit in flowers of multiple genera of Phyllanthaceae, including several species of *Kirganelia* (Kawakita & Kato, 2009; Kawakita et al., 2015; until recently in the genus *Phyllanthus*: Bouman et al., 2022). *K. microcarpa* (formerly *Phyllanthus microcarpus*) is a monoecious shrub (female flower, Figure 1c; male flower, Figure 1d) that grows along riverbanks or roadsides (Figure 1a,b) throughout tropical Asia (Luo et al., 2011). Three *Epicephala* species (*E. microcarpa*, *E. tertiaria*, *E. laeviclada*) with different ovipositor morphologies have previously been reported to be associated with this species (Li & Yang, 2015).

### 2.2 Pollination and oviposition behaviors

**FIGURE 2:**
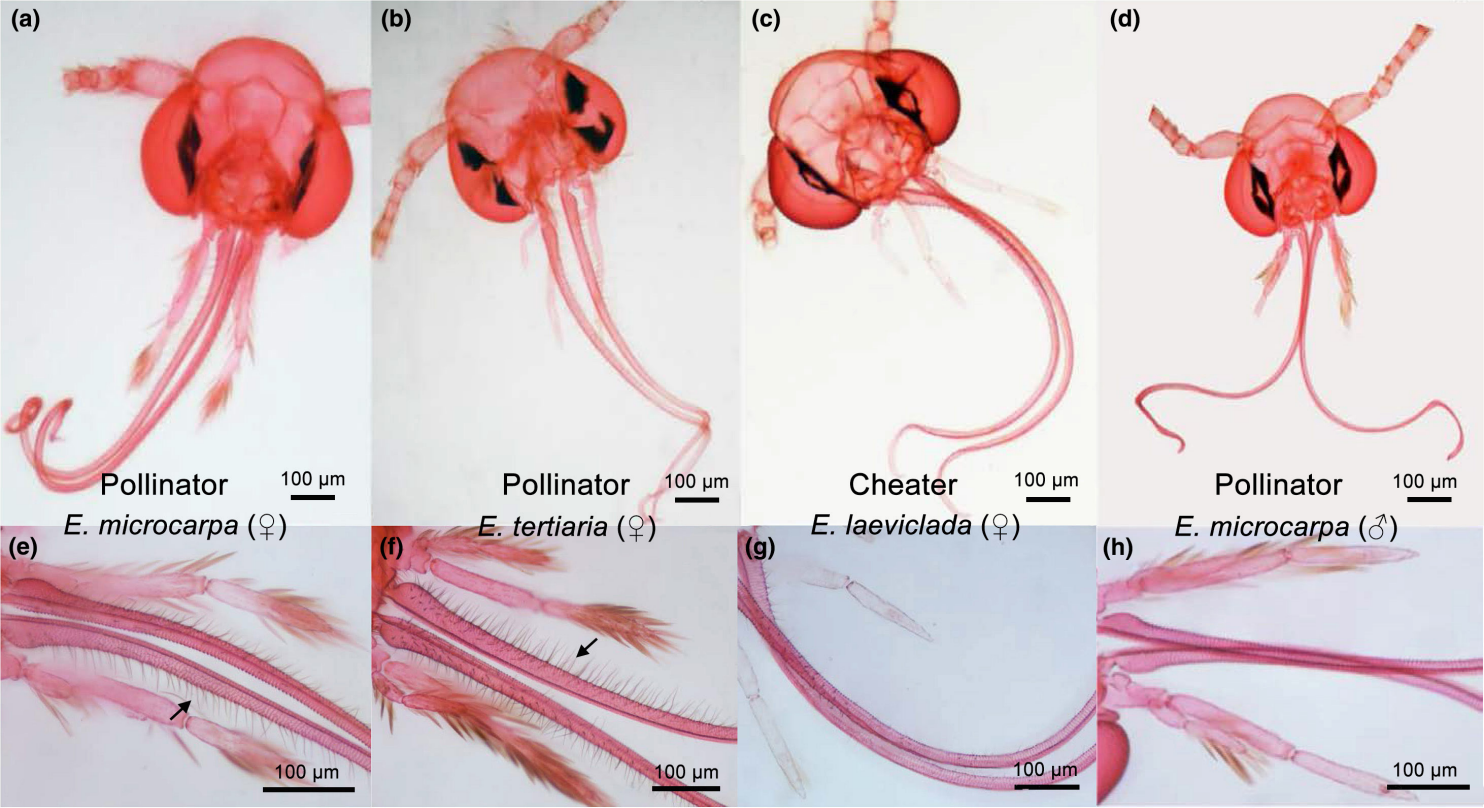
Morphology of head and proboscises of three species of *Epicephala* moths under a stereo microscope. Female moth (a) of the pollinator *E. microcarpa* with dense hairs on proboscis (e, black arrow); female moth (b) of the pollinator *E. tertiaria* with dense hairs on proboscis (f, black arrow); female moth (c) of cheater *E. laeviclada* with few short hairs on proboscis (g); male moth (d) of pollinator *E. microcarpa* without hairs on proboscis (h).

Our previous field observations in South China National Botanical Garden (Guangdong province), Huolushan Forest Park (Guangdong province), and Xinglong town (Hainan province) for more than 180 h in 3 years (2004–2006) suggested different *Epicephala* species among populations with different oviposition behaviors (see Luo, 2006). From 2016 to 2018, field observations were conducted again to investigate pollination and oviposition behaviors of *Epicephala* moths. For each moth, we observed and recorded the behaviors of inserting the proboscis into the stylar pit (pollination behaviors) and inserting the ovipositor into the stylar pit or laterally cutting through the ovary wall using the ovipositor (oviposition behaviors), and whether pollination occurred prior to oviposition. Each moth was collected after observation to identify the species based on morphology. In *Epicephala*, the presence of hairs on female proboscises is consistently predictive of pollination behavior (Kawakita & Kato, 2006, 2009). Consequently, we examined proboscis morphology to determine whether it was consistent with observed behavior: If the moth acts as a pollinator (with hairs) or cheater (without hairs). To do this, moth heads were removed and stored in a centrifuge tube with 10% KOH solution in a water bath heated at 75°C for 12 min, stained with Eosin dye solution, and mounted in Euparal (Waldeck) on glass slides under cover slips (Kawakita et al., 2015). The slides were then examined for the presence or absence of hairs on proboscises (Figure 2) under a stereoscope (Leica EZ4W).

### 2.3 Moth rearing and trait measurements

**FIGURE 3:**
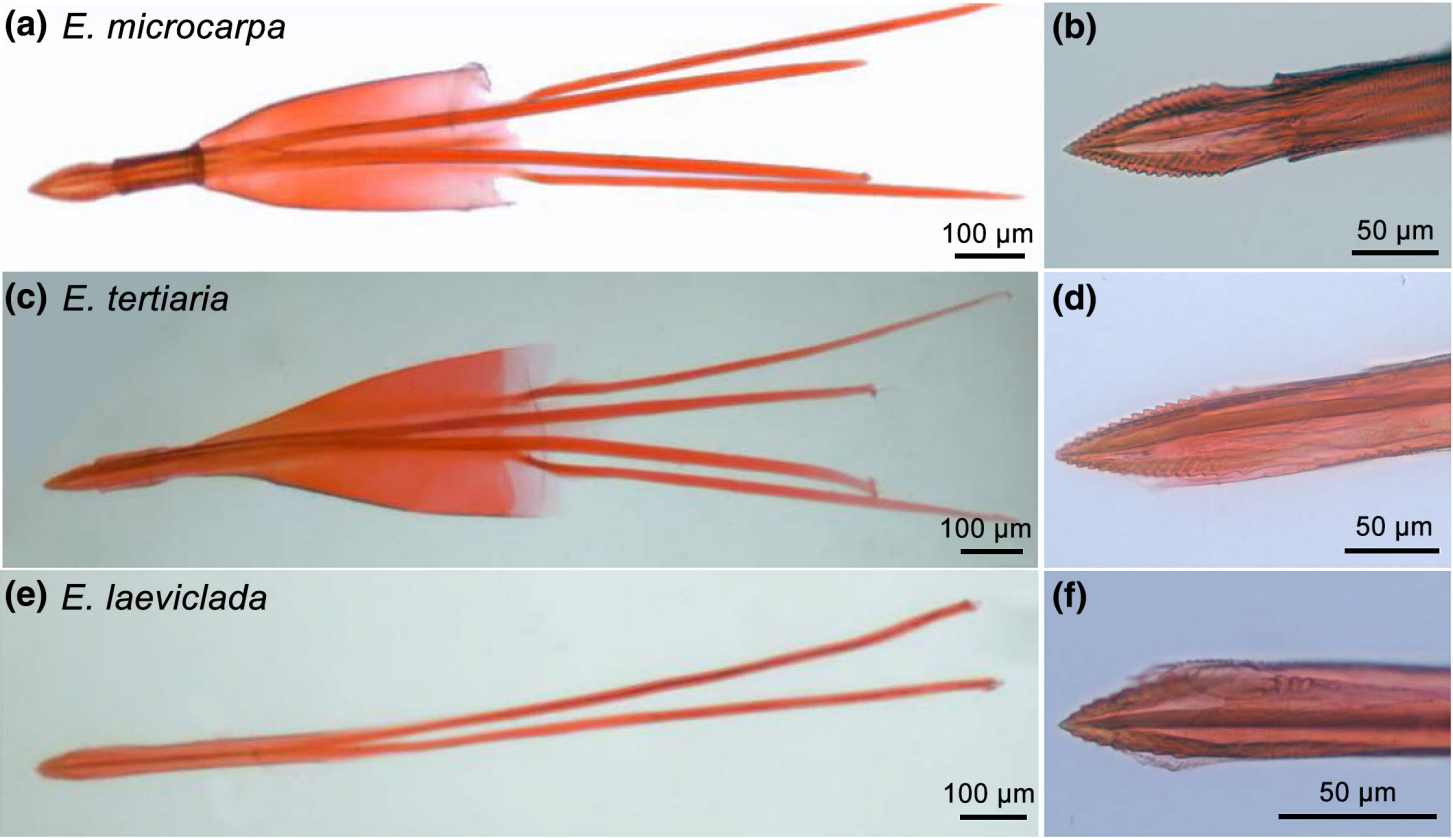
Morphology of ovipositor (a, c, e) and serrated keel on the tip of the ovipositor (b, d, f) of female *E. microcarpa*, *E. tertiaria* and *E. laeviclada*, respectively, stained with Eosin dye solution.

To investigate the spatial variation of *Epicephala* moth assemblages, immature fruits of *K. microcarpa* were collected from 16 sites of four provinces in China (Table 1) to rear larvae and adult moths. The fruits were placed in plastic food containers with wet tissue in the bottom to keep a certain air humidity. We recorded number and sexes of reared moths, then randomly dissected more than 10 moths (with equal numbers of males and females) from each site to identify the moth species based on morphology (Table 1). Additionally, forewing length (proxy for body size) and genitalia traits (Figures S2 and S3, male moth: saccus length, vinculum width, costa length, tegument length, phallus length; Figure 3 and Figure S3, female moth: ovipositor length, lamella antevaginalis length, apophysis anterioris length, apophysis posterioris length, keel length) were measured for each dissected moth. Both male and female genitalia were removed and stained with Eosin dye solution before measurements.

**TABLE 1:** Longitude, latitude, moth species assemblages, number of moths reared, number of moths dissected for species identification, number of plant individuals from which moths were reared, and number of plant individuals from which trait measurements were taken, for all 16 sites in this study.

Sites	Abbreviation	Longitude (E)	Latitude (N)	*Epicephala* moths	Reared moth number	Dissected moth number	Em:Et:El	Plant individuals	Sampled plant individuals
Jianfengling	JF	108°49′17″	18°42′51″	Em, Et, El	139 (69♂70♀)	14 (7♂7♀)	12:1:1	9	5
Diaoluoshan	DL	109°56′18″	18°46′05″	Em	90 (43♂47♀)	11 (5♂6♀)	11:0:0	4	2
Bawangling	BWL	109°05′47″	19°06′36″	Em, Et	133 (62♂71♀)	14 (7♂7♀)	5:9:0	10	4
Yinggeling	YG	109°34′12″	19°02′03″	Em, Et	118 (49♂69♀)	10 (5♂5♀)	9:1:0	4	2
Danzhou	DZ	109°31′13″	19°26′01″	Em, El	37 (14♂23♀)	11 (5♂6♀)	11:0:0	4	3
South China Botanical Garden	SCBG	113°21′31″	23°10′53″	Et, El	21 (5♂16♀)	15 (5♂10♀)	0:14:1	3	3
Wutongshan	WTS	114°11′32″	22°35′37″	Em, Et, El	18 (12♂6♀)	10 (5♂5♀)	10:0:0	3	1
Zhaoqing	ZQ	112°29′10″	23°06′32″	Em, Et, El	194 (104♂90♀)	12 (6♂6♀)	3:3:6	4	4
Baise	BS	106°33′54″	23°53′03″	Em, El	49 (18♂31♀)	10 (5♂5♀)	9:0:1	5	4
Dahua	DH	107°57′43″	23°44′53″	Em, Et, El	0	0		3	3
Pingxiang	PX	106°55′45″	22°03′04″	Em, Et, El	30 (17♂13♀)	10 (5♂5♀)	10:0:0	4	4
Zhubu	ZB	106°58′53″	22°34′39″	Em, Et	12 (6♂6♀)	10 (5♂5♀)	10:0:0	4	4
Naliang	NL	107°52′53″	21°40′36″	Em, Et	38 (15♂23♀)	12 (5♂7♀)	7:5:0	3	3
Nasuo	NS	108°06′31″	21°45′02″	Em, Et, El	10 (7♂3♀)	8 (5♂3♀)	5:3:0	4	3
Nanning	NN	108°22′10″	22°51′27″	Em, El	0	0		3	3
Xishuangbanna	BN	101°15′12″	21°55′50″	Em, Et	40 (15♂25♀)	11 (5♂6♀)	9:2:0	3	3

To investigate plant trait variation among populations, 30 leaves, 30 male flowers, and 30 female flowers were randomly sampled from 3 to 10 individuals (Table 1) for each site to measure leaf length, leaf width, stamen length, sepal length, stylar pit depth (distance from stigma to the placenta), and ovary wall thickness (Figure S4).

### 2.4 Data analyses

To examine whether ovipositor traits are correlated with other body traits, we conducted bivariate correlation analyses between forewing length (proxy for body size) and both male (phallus length) and female genitalia traits (ovipositor length, keel length). To estimate the degree of matching between ovipositor length and flower traits, we calculated mismatch index (MMI) with the equation MMI = (OL − FT)/FT, where OL represents moth ovipositor length and FT represents flower traits (stylar pit depth or ovary wall thickness). MMI = 0 means an exact match, −1 < MMI < 0 means the ovipositor is shorter than the flower traits, while 0 < MMI < 1 means the ovipositor is longer than the flower traits. The absolute value of MMI represents the degree of trait mismatching. To examine the trait matching between flowers and moths, generalized linear model analyses (GLMs) with a normal distribution and an identity function were carried out to compare the differences between stylar pit depth, ovary wall thickness, and ovipositor length of three *Epicephala* moths and to compare the differences of keel length among three moth species. To examine whether ovipositor traits match flower traits among different sites, bivariate correlation analyses were conducted between female flower traits (stylar pit depth and ovary wall thickness) and moth ovipositor traits (ovipositor length and keel length) for both *E. microcarpa* and *E. tertiaria*.

To examine whether flower traits and insect traits respond to different moth assemblages, pairwise contrast of GLMs with a normal distribution and an identity function were conducted to compare the differences of stylar pit depth and ovary wall thickness with moth assemblage as a treatment variable, that is, *K. microcarpa* populations associated with *E. microcarpa* alone (Em), in which *E. microcarpa* and *E. laeviclada* (Em‐l) coexisted, in which *E. microcarpa* and *E. tertiaria* (Em‐t) coexisted, in which *E. microcarpa*, *E. tertiaria*, and *E. laeviclada* (Em‐t‐l) coexisted, and in which *E. tertiaria* and *E. laeviclada* (Et‐l) coexisted. Additionally, pairwise contrast of GLMs with a normal distribution and an identity function were conducted to compare differences of ovipositor length and keel length of *E. microcarpa* moths which coexisted with *E. tertiaria* or/and *E. laeviclada*, and the differences in ovipositor length and keel length of *E. tertiaria* moths which coexisted with *E. microcarpa* or/and *E. laeviclada*, respectively.

All analyses were conducted in SPSS v. 22.0 (IBM Inc.), all histogram, and scattergram were made in Origin v. 9.0 (OriginLab Inc.), and all data were logarithmically transformed to be normally distributed before analysis.

## 3 RESULTS

### 3.1 Pollination and oviposition behaviors

The three *Epicephala* moth species differed in proboscis morphology and oviposition behaviors. Female moths of both *E. microcarpa* and *E. tertiaria* have dense long hairs on their proboscises (Figure 2e,f) but only a few short hairs occurred on the proboscis of female *E. laeviclada* (Figure 2g). Pollination behaviors by inserting proboscis into the stylar pit were indeed observed in these *Epicephala* species (Figure 1e,g) except *E. laeviclada*. Additionally, hairs were not detected on the male moths of *E. microcarpa* (Figure 2h), suggesting that only female moths provided pollination services (consistent with other species of *Epicephala*: Kawakita & Kato, 2006; Kawakita et al., 2015). In the field, we observed that female *E. tertiaria* moths laid eggs by inserting the ovipositor into the flower's stylar pit and laying eggs (stylar pit oviposition; Figure 1h,i), but female *E. microcarpa* and *E. laeviclada* laid eggs by laterally cutting through the ovary wall with the ovipositor and depositing eggs within the locule (locular oviposition; Figure 1f). These results suggested that both *E. microcarpa* and *E. tertiaria* moths served as seed‐predating pollinators while *E. laeviclada* served as a cheater (or third‐party exploiter) in the *K. microcarpa‐Epicephala* system. Notably, we saw no evidence that *E. laeviclada* galls host fruit, unlike several undescribed non‐mutualistic *Epicephala* reported from *Kirganelia* in Taiwan (Kawakita et al., 2015).

### 3.2 Spatial pattern of *Epicephala* moth coexistence

**FIGURE 4:**
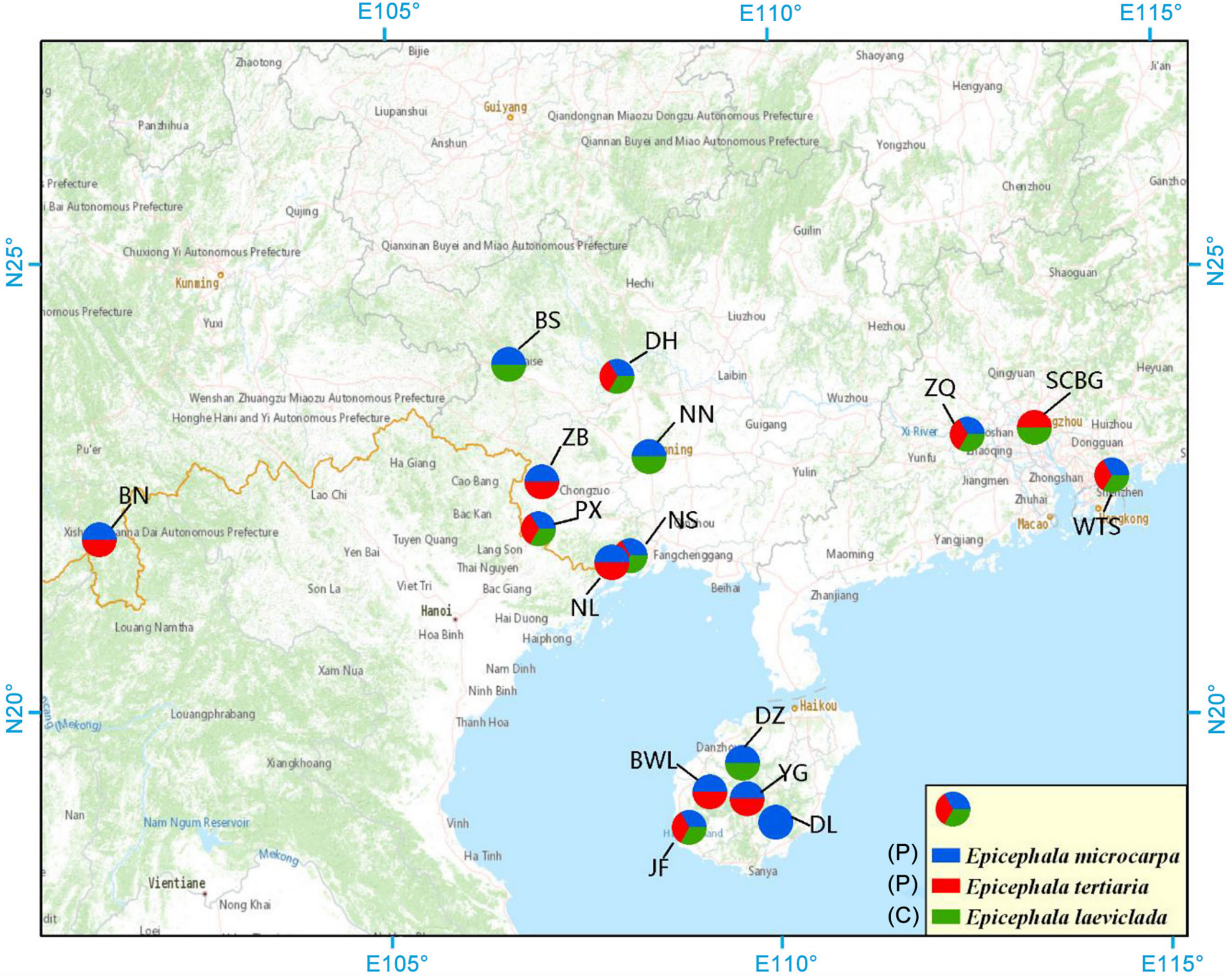
Locations of the 16 studied sites, indicating which three *Epicephala* moths (blue: *E. microcarpa*; red: *E. tertiaria*; green: *E. laeviclada*) coexist in local assemblages at each site. The letters in brackets indicate moth pollinator (P) or cheater (C).

Our field observations and rearing results showed that multiple *Epicephala* moth species coexisted at all studied populations except Diaoluoshan (Figure 4; Table 1). Specifically, all three moth species coexisted (Em‐t‐l) at six populations, *E. microcarpa* and *E. tertiaria* coexisted (Em‐t) at five populations, *E. microcarpa* and *E. laeviclada* coexisted (Em‐l) at three populations, *E. tertiaria* and *E. laeviclada* coexisted (Et‐l) at only one population. At all studied populations, *E. microcarpa* was the dominant visitor: It had both the widest distribution (occurring at 15 out of 16 studied populations) and the highest abundance of the three moth species (Figure 4; Table 1). The cheater *E. laeviclada* occurred at 10 out of 16 populations and always coexisted with pollinators (Figure 4; Table 1), suggesting geographic variation in moth assemblages associated with *K. microcarpa*.

### 3.3 *Epicephala* ovipositor morphology associated with floral traits

Correlation analyses showed that phallus length was significantly positively correlated with forewing length in both *E. microcarpa* and *E. tertiaria* male moths (Table 2; *r* = .875, *p* < .001; *r* = .498, *p* < .001, respectively); however, both ovipositor length and keel length did not show significant correlations with forewing length in all three *Epicephala* female moths (Table 2; all *p* > .05), suggesting that ovipositor traits evolved under selective pressures from external factors and are not simply correlated with female body size.

**TABLE 2:** Pearson's correlation values (left) and *p*‐values (right) between forewing length (proxy for body size) and genitalia traits (female: ovipositor and keel length; male: phallus length) based on bivariate correlation analyses in three *Epicephala* moth species. Bold values indicate significant correlations at *p* < .05. The letters in brackets indicate pollinator (P) or cheater (C), and the values in brackets indicate sample sizes.

Sex	Traits	Forewing length		
		*E. tertiaria* (P)	*E. microcarpa* (P)	*E. laeviclada* (C)
Female	Ovipositor length	.144/.557 (19)	.187/.161 (58)	.590/.410 (4)
	Keel length	−.149/.543 (19)	.161/.228 (58)	.854/.146 (4)
Male	Phallus length	**.875**/**<.001 (15)**	**.498**/**<.001 (54)**	.519/.233 (7)

**FIGURE 5:**
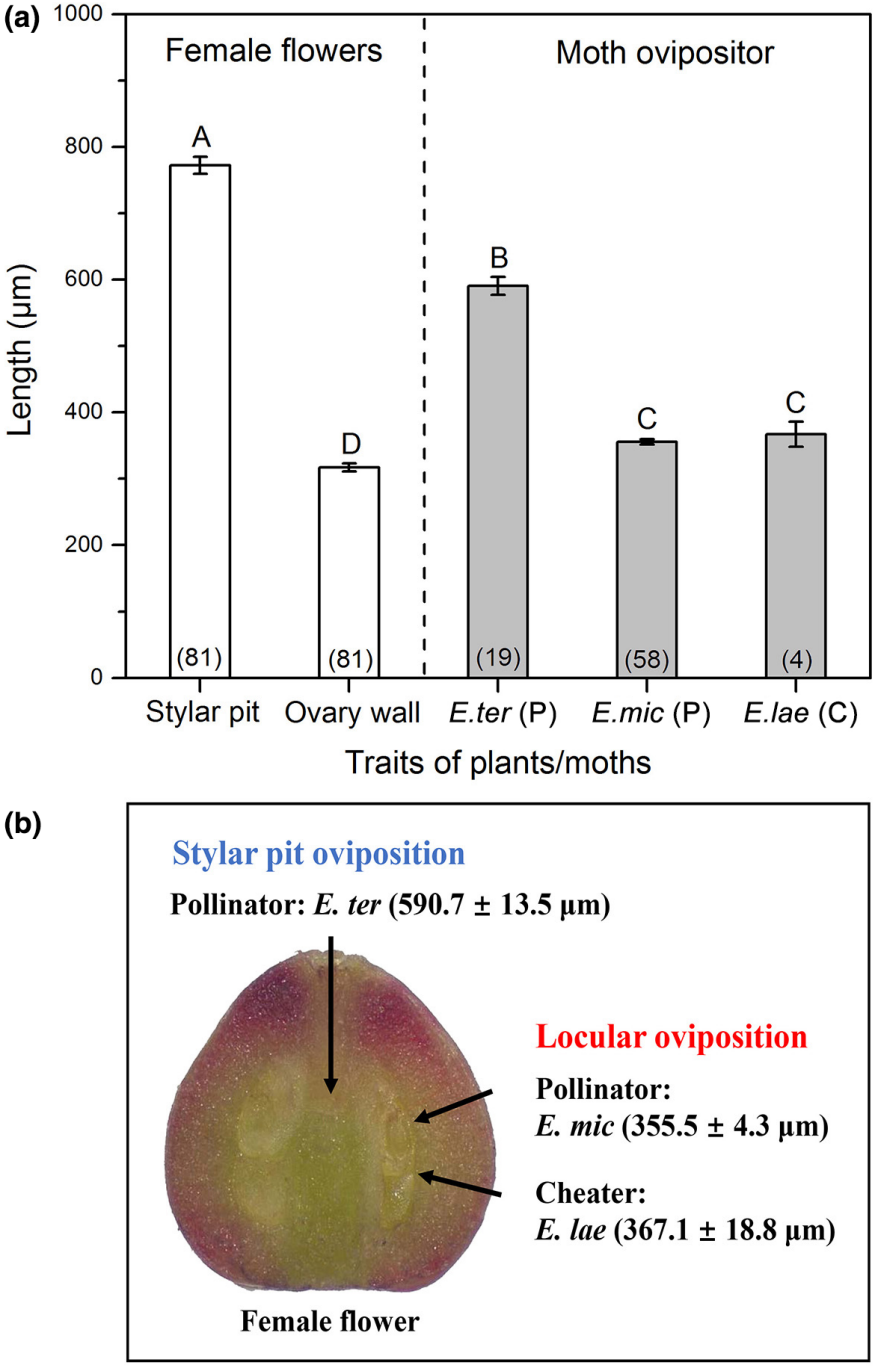
(a) Comparisons of female flower traits (mean ± SE; white bars; stylar pit depth and ovary wall thickness) and ovipositor length of three *Epicephala* species (gray bars) under generalized linear model analyses. Different letters above the bars indicate significant differences at *p* < .05, and the values in brackets indicate sample sizes. Proboscis tip morphology of the three moth species are shown above the bars, respectively. (b) Trait matching of ovipositor length (mean ± SE) to different oviposition behaviors (blue arrow: stylar pit oviposition; red arrows: locular oviposition) in three *Epicephala* species.

**FIGURE 6:**
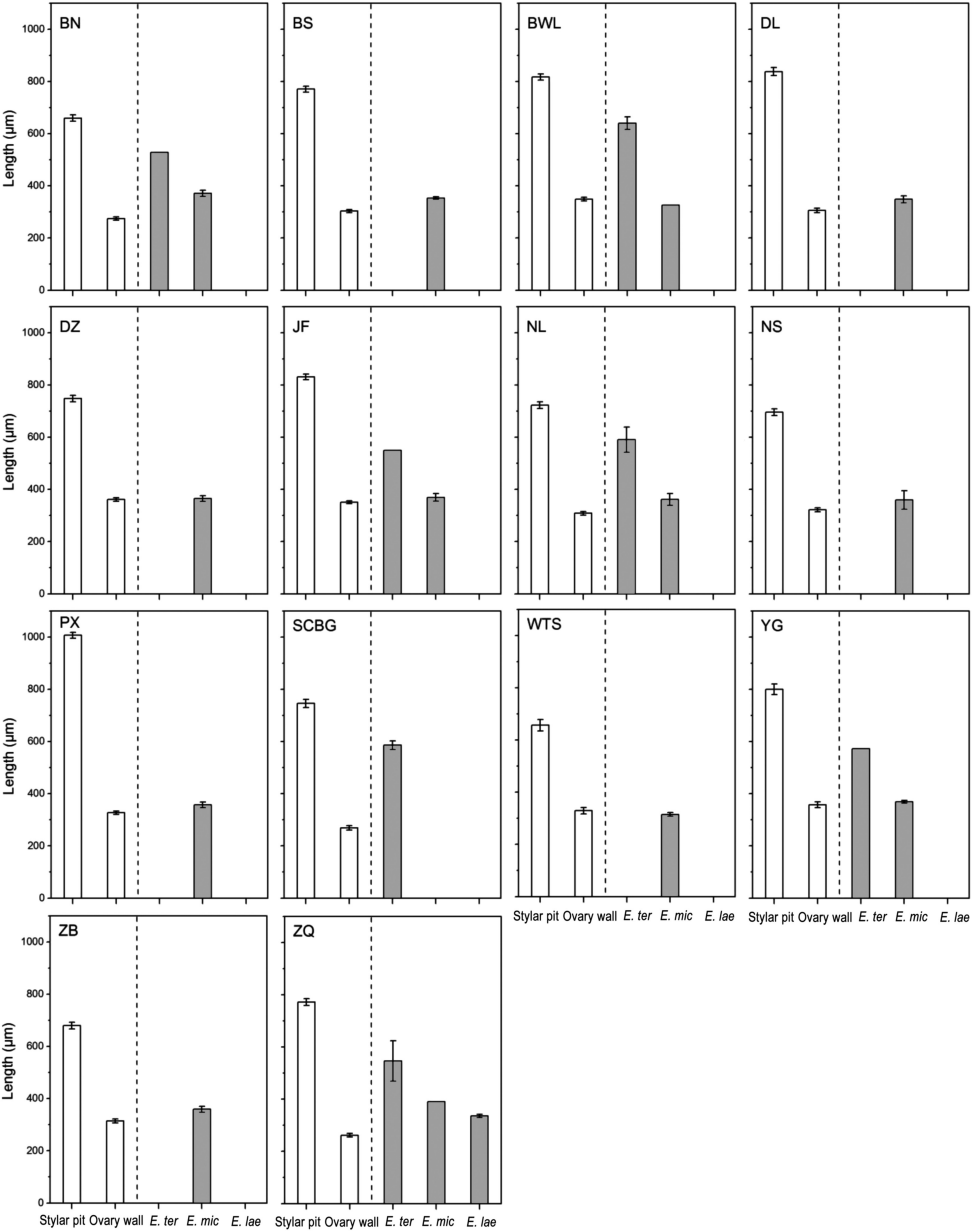
Floral traits (mean ± SE; white bars) and ovipositor length (gray bars) of the three *Epicephala* moth species at the 14 studied sites. In each population, 5–7 female moths were randomly sampled to measure ovipositor length, resulting in only one individual from certain moth species in some plant populations. Bars lacking error bars indicate those moth species for which only one individual was measured as a result of the sampling regime.

**FIGURE 7:**
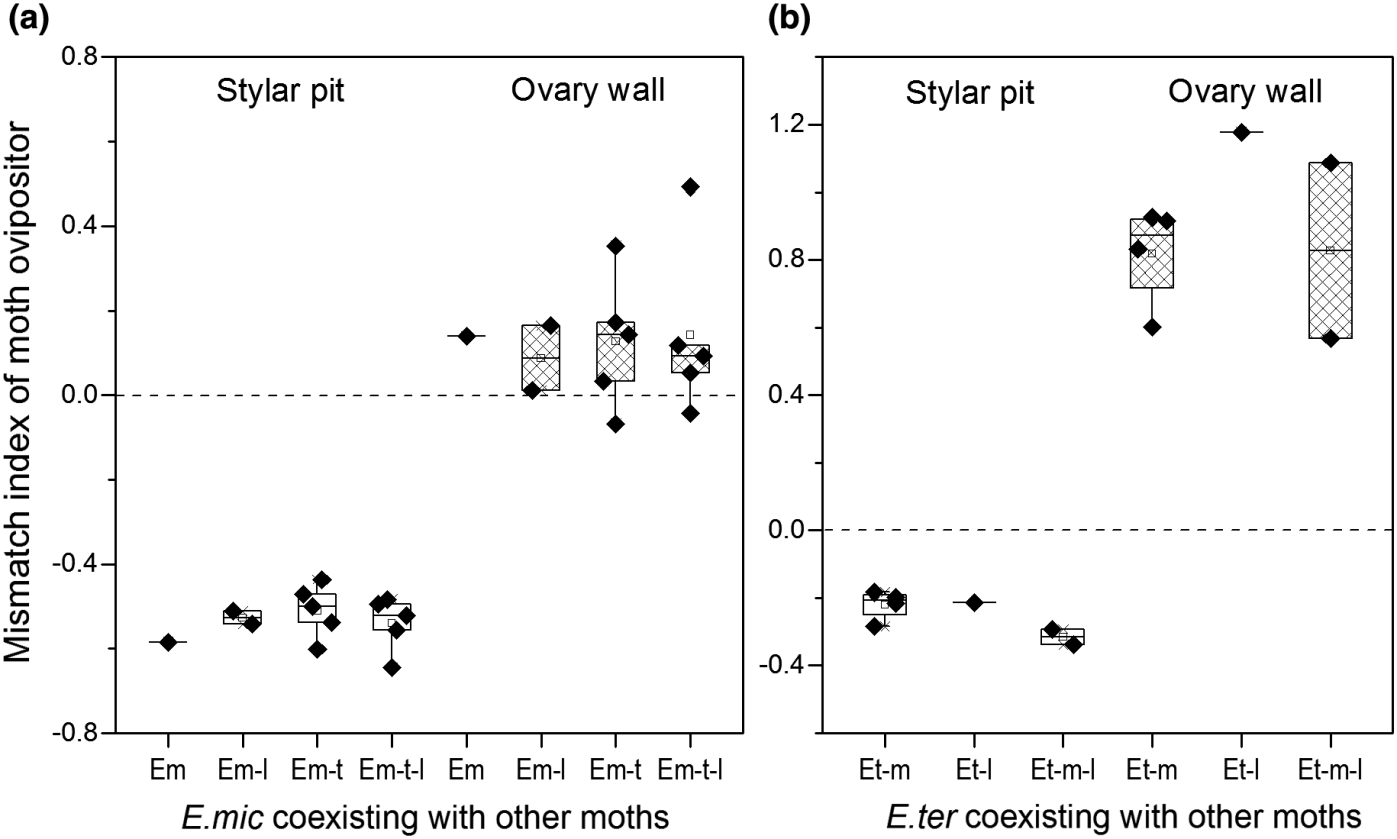
Mismatch index between moth ovipositor length and floral traits (stylar pit depth, open boxes; ovary wall thickness, grid boxes) among populations with different moth assemblages. The dashed lines (*y* = 0) indicate exact match between ovipositor and floral traits. (a) Pollinator *Epicephala microcarpa*; (b) pollinator *E. tertiaria*.

Results of generalized linear models (GLMs) showed that ovipositor length was significantly longer (all *p* < .001) in *E. tertiaria* (mean ± SE = 590.7 ± 13.5 μm) than that in *E. microcarpa* (355.5 ± 4.3 μm) and *E. laeviclada* (367.1 ± 18.8 μm) (Figure 5a), while the latter two species did not show significant difference from each other (*p* = .766; Figure 5a), indicating that ovipositor length differed among moth species with different oviposition behaviors. Although comparison analyses showed significant differences between female flower traits (stylar pit depth or ovary wall thickness) and ovipositor length (Figure 5a), the mismatch index results suggested that ovipositor length of *E. microcarpa* and *E. laeviclada* more closely matched ovary wall thickness (Table 4; mismatch index = 0.121 and 0.157, respectively), while ovipositor length of *E. tertiaria* more closely matched stylar pit depth (Table 4; mismatch index = −0.244) at species level. Similarly, these patterns were seen globally as well as in each studied population (Figure 6; Table 5) and among populations with different moth assemblages (Figure 7).

**FIGURE 8:**
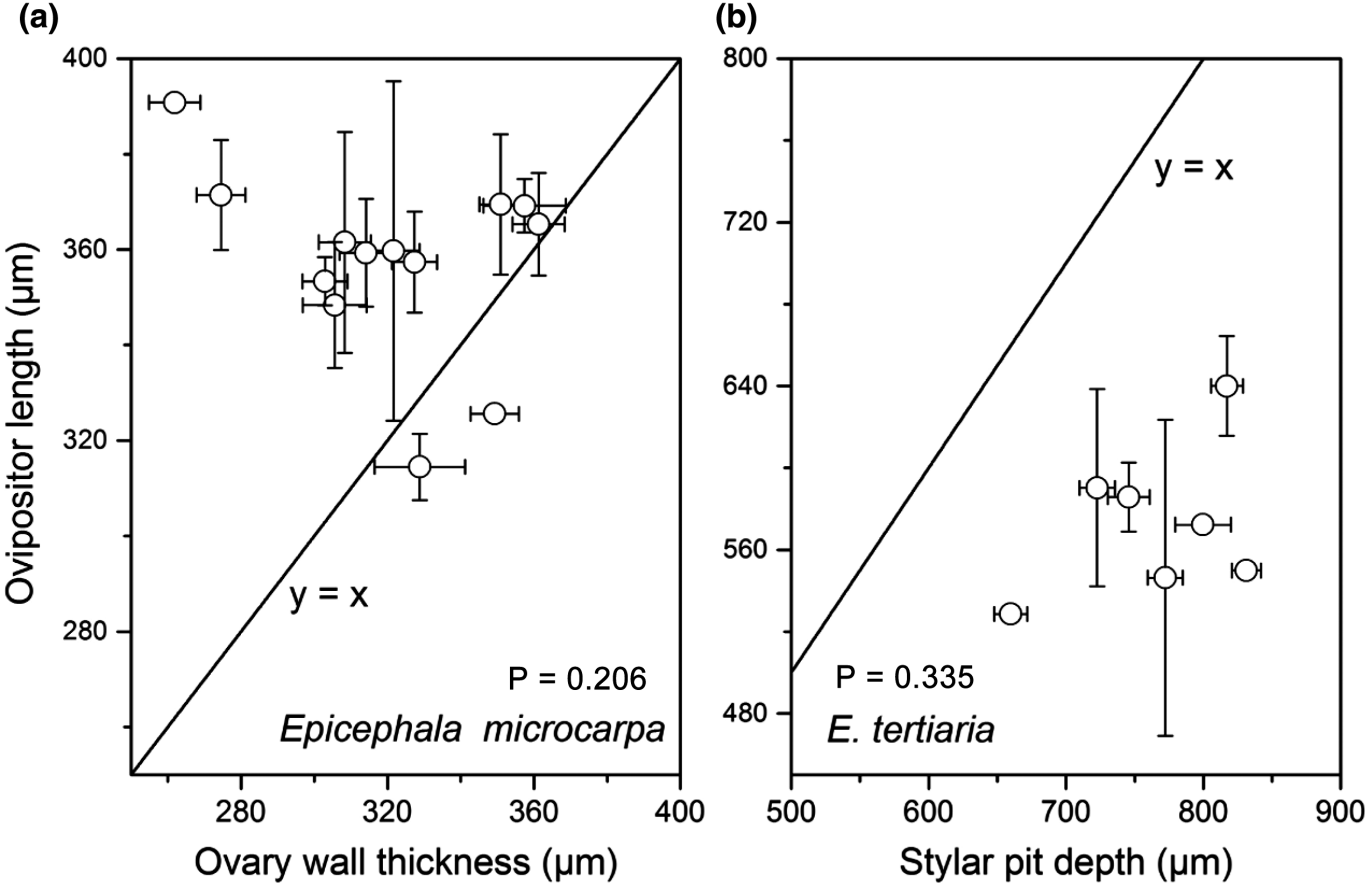
Trait matching between floral traits and ovipositor length among studied sites. (a) Ovipositor length of *E. microcarpa* and ovary wall thickness; (b) ovipositor length of *E. tertiaria* and stylar pit depth. The solid lines (*y* = *x*) indicate an exact match between flower and insect traits. *p*‐Value indicates significant correlation at *p* < .05.

Moreover, our correlation analyses between female flower traits and ovipositor traits among different populations showed that both ovipositor length and keel length were not significantly correlated (all *p* > .05) with stylar pit depth or ovary wall thickness, either in *E. microcarpa* or *E. tertiaria* (Table 3; Figure 8). Additionally, GLMs results showed significant differences (Wald *χ* ^2^ = 2.863, *p* < .001) in keel length among the three moth species, with the longest in *E. microcarpa* (96.8 ± 1.1 μm), intermediate lengths in *E. tertiaria* (90.1 ± 3.8 μm), and the shortest in *E. laeviclada* (52.1 ± 2.2 μm), although the functional significance of keel length remains unknown.

**TABLE 3:** Pearson's correlation values (upper right) and *p*‐values (lower left) between female flower traits (stylar pit depth, ovary wall thickness) and ovipositor traits (ovipositor length, keel length) of two *Epicephala* moth pollinator species among 16 studied populations.

Moth pollinator	Stylar pit	Ovary wall	Ovipositor	Keel
*E. microcarpa*				
Stylar pit		.378	.098	.062
Ovary wall	.149		−.375	−.216
Ovipositor	.751	.206		.300
Keel	.840	.479	.319	
*E. tertiaria*				
Stylar pit		.378	.425	.604
Ovary wall	.877		.430	.120
Ovipositor	.335	.797		.073
Keel	.149	.342	.151	

### 3.4 Trait variations in response to moth assemblages

**FIGURE 9:**
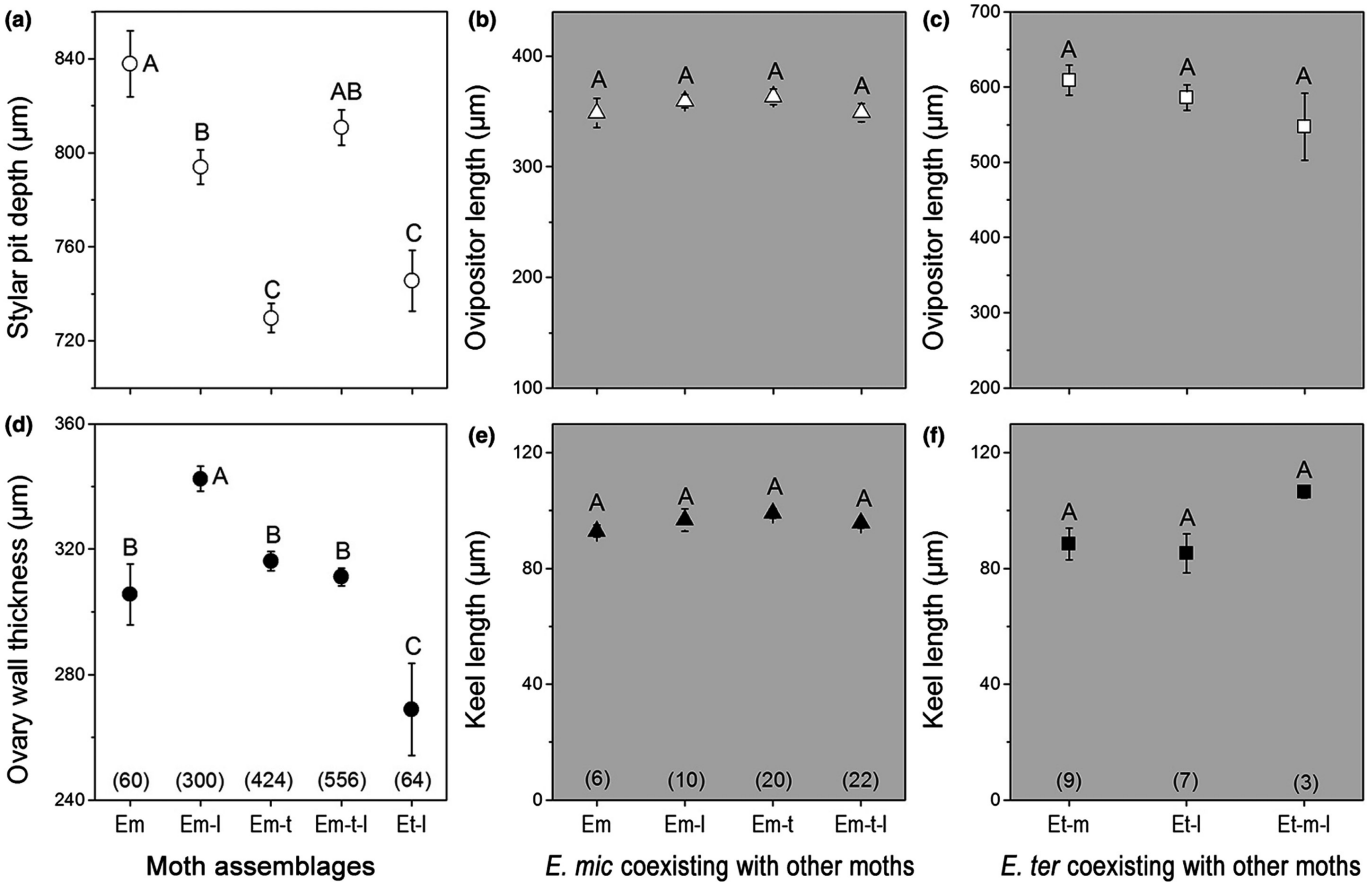
Comparisons of stylar pit depth (a; mean ± SE) and ovary wall thickness (d), respectively, associated with different *Epicephala* moth assemblages under generalized linear model analyses. Comparisons of ovipositor length (b) and keel length (e) of *E. microcarpa* when it coexisted with different moth species. Comparisons of ovipositor length (c) and keel length (f) of *E. tertiaria* when it coexisted with different moth species. Moth assemblages: Em = represents *E. microcarpa* alone; Em‐l = coexistence of *E. microcarpa* and *E. laeviclada*; Em‐t = coexistence of *E. microcarpa* and *E. tertiaria*; Em‐t‐l = coexistence of *E. microcarpa*, *E. tertiaria*, and *E. laeviclada*; Et‐l = coexistence of *E. tertiaria* and *E. laeviclada*. Different letters above the bars indicate significant differences at *p* < .05, and the values in parentheses indicate sample sizes.

Generalized linear model analyses results showed that both ovipositor and keel length of *E. microcarpa* were not significantly different when *E. microcarpa* coexisted with *E. tertiaria* or/and *E. laeviclada* (Wald *χ* ^2^ = 2.863, *p* = .413; Wald *χ* ^2^ = 2.261, *p* = .447, respectively; Figure 9b,e). Similarly, the results showed no significant differences in ovipositor and keel length in *E. tertiaria* when it coexisted with *E. microcarpa* or/and *E. laeviclada* (Wald *χ* ^2^ = 2.849, *p* = .241; Wald *χ* ^2^ = 3.650, *p* = .161, respectively; Figure 9c,f), suggesting that ovipositor traits do not respond evolutionarily to the presence of other moth species.

However, both stylar pit depth and ovary wall thickness of *K. microcarpa* female flowers were significantly different in the presence of different *Epicephala* species assemblages (Wald *χ* ^2^ = 92.129, *p* < .001; Wald *χ* ^2^ = 93.478, *p* < .001, respectively; Figure 9a,d). Specifically, the ovary wall thickness of flowers in populations with only *E. microcarpa* (Em; 305.6 ± 9.7 μm) was significantly thinner (*p* < .001) than in populations with both *E. laeviclada* and *E. microcarpa* (Em‐l; 342.5 ± 4.0 μm) (Table 6; Figure 9d) but was not significantly different (*p* = .139) than in populations with both *E. tertiaria* and *E. microcarpa* (Em‐t; 316.2 ± 3.0 μm) (Figure 9d), suggesting that the occurrence of cheater *E. laeviclada* (which has locular oviposition) in a population would induce mechanical defense (thicker ovary wall) by the host plants. Moreover, the ovary wall was significantly thicker (*p* = .003) in plant populations associated with all three moths (Em‐t‐l; 311.2 ± 2.8 μm) than in plant populations with both *E. tertiaria* and *E. laeviclada* (Et‐l; 268.9 ± 14.6 μm) (Table 6; Figure 9d), suggesting that mechanical defense of plants responded to locular oviposition behaviors, whether by the pollinator or cheater.

On the contrary, flower stylar pits were significantly shallower (*p* = .022; *p* < .001; respectively) in flowers from populations with *E. microcarpa* and *E. tertiaria* (Em‐t) than those from populations with *E. microcarpa* alone (Em) and or with *E. microcarpa* and *E. laeviclada* (Em‐l) (Table 6; Figure 9a), but showed no significant difference (*p* = .276) from flowers from populations with *E. tertiaria* and *E. laeviclada* (Et‐l) (Figure 9a), suggesting that stylar pits tend to be shallower and a closer trait match to the pollinator *E. tertiaria* where it was present. In addition, stylar pits were significantly deeper (*p* = .003) in flowers from populations with all three moth species (Em‐t‐l) than from populations with only *E. tertiaria* and *E. laeviclada* (Et‐l) (Table 3; Figure 9a), for reasons that remain unclear.

## 4 DISCUSSION

### 4.1 Trait matching in situations with multiple partners

Three species of *Epicephala* moths—*E. tertiaria*, *E. microcarpa* and *E. laeviclada*—laid eggs in female flowers of the leafflower *Kirganelia microcarpa* but exhibited different oviposition behaviors: stylar pit oviposition by *E. tertiaria* and locular oviposition by *E. microcarpa* and *E. laeviclada*. *Epicephala tertiaria* and *E. microcarpa* provided active pollination services with ciliated proboscises before ovipositing (and thus acted as pollinators) while *E. laeviclada* lacked proboscis hairs and was not observed to pollinate the flowers (and thus acted as a cheater). The cheater *E*. *laeviclada* occurred in most populations (10/16) and always coexisted with pollinators, but which pollinators were present varied among different populations. Ovipositors of female moths of the three species differed in both length and morphology, which appeared functionally suited to different oviposition behaviors and exhibited some trait matching with stylar pit depth (in *E. tertiaria*) and ovary wall thickness (in *E. microcarpa* and *E. laeviclada*). However, our analyses did not show significant correlations between flower and ovipositor traits among populations. Additionally, flower trait variations responded to different local assemblages of moth species in ways that seemed to suggest antagonistic responses to the locule ovipositors *E. macrocarpa* and *E. laeviclada* but, oddly, mutually beneficial responses to the stylar pit ovipositor *E. tertiaria*.

Coevolution and mutualism may shape reciprocal selection and coadaptation between partners, especially in specialized plant‐pollinator systems where the match between plant and insect traits will affect pollen deposition and plant fitness (Muchhala & Thomson, 2009). Similarly, trait matching between ovipositor length and floral traits should be favored for oviposition behaviors in specialized seed‐predating pollination systems, although this may be due either to mutualistic or antagonistic coevolution (Godsoe et al., 2010; Yang & Li, 2018). In this study, three *Epicephala* moth species laid eggs in female flowers using diverse oviposition behaviors, and our results suggested a match between a longer ovipositor and flower stylar pit depth in *E. tertiaria* performing stylar pit oviposition (Figure 5a,b; Table 4), and a match between short ovipositors and ovary wall thickness in *E. microcarpa* and *E. laeviclada* performing locular oviposition (Figure 5a,b; Table 4). These results support the hypothesis that moth ovipositor traits are adapted to specific flower traits, depending on the oviposition behavior of the moth species. Additionally, our results suggested trait matching between floral traits and ovipositor length for both pollinators (*E. microcarpa* and *E. tertiaria*) and cheater (*E. laeviclada*) at species level, which is consistent with the positive correlations between style length/wall width and pollinator/non‐pollinator ovipositor length in fig–fig wasp mutualism (Weiblen, 2004).

**TABLE 4:** Mismatch index (MMI) between ovipositor length of three *Epicephala* moth species and female flower traits (stylar pit depth and ovary wall thickness). MMI = 0 means an exact match, MMI < 0 means the ovipositor is shorter than the flower trait, and MMI > 0 means the ovipositor is longer than the flower trait. The absolute value of MMI represents the degree of trait mismatch.

Mismatch index (MMI)	Ovipositor length		
	*E. laeviclada* (367.1 μm)	*E. microcarpa* (355.5 μm)	*E. tertiaria* (590.7 μm)
Stylar pit depth (772.4 μm)	−0.525	−0.540	−0.235
Ovary wall thickness (317.4 μm)	0.157	0.120	0.861

In other studies, functional traits of interacting partners, such as nectar spur length of flowers and pollinator proboscis length in pollination mutualisms (Darwin, 1862), or cone structure and bill size in seed dispersal interactions (Benkman et al., 2003), have been shown to be strongly correlated among different sites due to stabilizing selection from a primary partner species, which results in a coevolutionary arms race. Empirical evidence from the pollination mutualism between long‐tongued flies and the plants *Zaluzianskya microsiphon* (Scrophulariaceae) and *Lapeirousia anceps* (Iridaceae) supports this hypothesis (Anderson & Johnson, 2008; Pauw et al., 2009). However, whether such arms races can explain trait matching when partners are part of multi‐species assemblages with various relevant trait values is less clear (Haber & Frankie, 1989; Harder, 1985). In a study of an oil‐producing flower and three species of oil‐collecting bees, Hollens‐Kuhr et al. (2021) found that among‐population variation in the degree of trait matching that had an inverse relationship to pollinator community diversity. In our study, since the three *Epicephala* moths laid eggs in *K. microcarpa* via different oviposition behaviors, and two of them pollinated flowers by inserting the proboscis into the stylar pit, it seems plausible that plant traits might evolve under balancing selection from three partners and two interactions (pollination and oviposition). As predicted, stylar pit depth and ovary wall thickness showed relatively lower variations and higher contributions to the primary principal component compared to male flower traits and leaf traits (Tables S1 and S2). Additionally, our results indicated that stylar pit depth was less in populations with *E. tertiaria* as the primary pollinator and greater in populations with *E. microcarpa* (Table 6), suggesting that plants may be responding to pollinator oviposition behaviors, although there were no significant correlations between ovipositor length and floral traits among different populations (Table 2). Since all three moths exhibited close trait matching of ovipositor length to floral traits at species level, the lack of covariation between functional traits at the population level might reflect the effects of selection from multiple partners, as well as among‐population gene flow (Yoder et al., 2013).

The geographic mosaic theory of coevolution (GMTC; Thompson, 2005) predicts that at the metapopulation level, interacting partners may in some populations show both trait matching (hot spots) and trait mismatching (cold spots) (Gomulkiewicz et al., 2000; Thompson, 1999). Although we know that evaluating the selection is important for testing GMTC, data are not available for us to do this analysis in the present study. Our MMI results between floral traits and ovipositor length showed great variation among 16 sites (Figure 7; Table 5), ranging from 0.011 (close match) to 0.492 (poor match) between ovary wall thickness and ovipositor length of *E. microcarpa* and from 0.183 to 0.338 between stylar pit depth and ovipositor length of moth *E. tertiaria* in consistent with predictions from geographic mosaic theory, suggesting relaxed matching of ovipositor length to flower traits which might be due to arms‐race dynamics or genetic constraints. Because of the dominant (14 out of 16) positive MMI values in all populations (Figure 7; Table 5), which means the ovipositor length of *E. microcarpa* was usually greater than the ovary wall thickness and thus potentially adapted to locular oviposition, it seems that the moths can track the changes in host plant tissue depth. As the only two previous studies of trait matching among populations in brood pollination mutualism, Godsoe et al. (2010) found trait matching between stylar canal length and ovipositor length at the species level but no indication of trait matching at the population level in yucca–yucca moth interactions, and Xiao et al. (2017) observed positive correlations between fig wall thickness and fig wasp ovipositor length among six populations. Similarly, our results showed a close match between *K. microcarpa* and three *Epicephala* moths at the species level (Figure 5a; Table 4) and population level (Figure 7), with varied MMI among populations. All these studies suggest the potential for complicated coadaptation of functional traits in brood pollination mutualism, under the balancing selection between pollination and predation, and (in this study) within multi‐species assemblages.

**TABLE 5:** Mean values of floral traits (stylar pit depth and ovary wall thickness), ovipositor length of three *Epicephala* species, and the mismatch index (MMI) between flower and insect traits at 16 studied sites. MMI = 0 means an exact match, MMI < 0 means the ovipositor is shorter than the flower trait, and MMI > 0 means the ovipositor is longer than the flower trait. The absolute value of MMI represents the degree of trait mismatch. The letters in brackets indicate moth pollinator (P) or cheater (C).

Sites	*Epicephala* moths	Floral traits		*Epicephala microcarpa* (P)			*E. tertiaria* (P)			*E. laeviclada* (C)		
		Stylar pit depth (μm)	Ovary wall thickness (μm)	Ovipositor length (μm)	MMI (stylar pit)	MMI (ovary wall)	Ovipositor length (μm)	MMI (stylar pit)	MMI (ovary wall)	Ovipositor length (μm)	MMI (stylar pit)	MMI (ovary wall)
DL	Em	837.9	305.6	348.4	−0.584	0.140						
BS	Em‐l	770.4	302.9	352.6	−0.542	0.164						
DZ	Em‐l	747.8	361.3	365.3	−0.511	0.011						
NN	Em‐l	871.3	376.3									
BN	Em‐t	659.7	274.5	371.5	−0.437	0.353	528.6	−0.199	0.926			
BWL	Em‐t	817.1	349.3	325.6	−0.601	−0.068	640.0	−0.217	0.832			
NL	Em‐t	722.6	308.3	361.5	−0.500	0.172	590.3	−0.183	0.915			
YG	Em‐t	799.8	357.4	369.2	−0.538	0.033	572.2	−0.284	0.601			
ZB	Em‐t	679.3	314.2	359.3	−0.471	0.144						
DH	Em‐t‐l	723.9	264.6									
JF	Em‐t‐l	831.2	350.9	369.4	−0.556	0.053	549.9	−0.338	0.567			
NS	Em‐t‐l	695.8	321.7	359.7	−0.483	0.118						
PX	Em‐t‐l	1006.6	327.4	357.4	−0.645	0.092						
WTS	Em‐t‐l	656.8	328.8	314.5	−0.521	−0.043						
ZQ	Em‐t‐l	772.2	261.9	390.8	−0.494	0.492	546.3	−0.293	1.086	335.3	−0.566	0.280
SCBG	Et‐l	745.6	268.9				585.7	−0.214	1.178			

### 4.2 Trait variations in response to different interacting partners

In mutualisms, interacting partners are expected to evolve mechanisms to limit excessive mutual exploitation, thus maintaining the mutualism (Pellmyr & Huth, 1994). Partner choice, host sanctions, and partner fidelity feedback are among the mechanisms that have been hypothesized to function to maintain mutualism (Bull & Rice, 1991; Leigh, 2010; Weyl et al., 2010). In this study, we examined how local moth species assemblages affect the covariation of functional traits in both female flowers and moth ovipositors, which may have implications for mutualism stability. Our results indicate that both ovipositor length and keel length in *E. microcarpa* and *E. tertiaria* did not respond to coexistence with other moth species, while flower stylar pit depth and ovary wall thickness varied significantly under different moth species assemblages (Figure 9a,d). Since *Epicephala* larvae consume seeds of their host *K. microcarpa*, oviposition increases moth reproductive fitness but decreases host plant fitness. As a result, plants might have evolved defense mechanisms in response to oviposition behaviors. Here, our results showed that ovary wall thickness significantly increased when the cheater *E. laeviclada* existed in the populations (Figure 9d; Table 6). Similarly, this increase also occurred when the pollinator *E. microcarpa* existed (Figure 9d; Table 6), which means that the plants might increase ovary wall thickness to defend against locular oviposition regardless of the ecological role of the insects (pollinator or cheater). However, the ovary wall thickness of flowers in populations with all three moths showed no significant difference (*p* = .173) with that of flowers from populations with only *E. microcarpa* and *E. tertiaria* (Em‐t; Table 6) and was significantly thinner (*p* < .001) than that of flowers from populations with only *E. microcarpa* and *E. laeviclada* (Em‐l; Figure 9d), for reasons that remain unclear. In addition, ovary wall thickness was always higher in the presence of *E. microcarpa* relative to when *E. microcarpa* was absent (Figure 9d), indicating that *E. microcarpa* is probably the main factor determining variation in ovary wall thickness. This pattern is probably due to the high abundance of *E. microcarpa* in most plant populations (Table 1).

**TABLE 6:** Effects of different *Epicephala* moths and behaviors on floral trait (stylar pit depth and ovary wall thickness) variations. Bold values indicate significant differences at *p* < .05 under generalized linear model analyses. The letters in brackets indicate moth pollinator (P) or cheater (C). The symbol “**↓**” indicates decreasing trends, “**↑**” indicates increasing trends, and “**−**” indicates no significant variation.

Floral traits	Factors	Related behaviors	Moth assemblages		Trends	*p*
Stylar pit depth	*E. tertiaria* (P)	Pollination and Oviposition	Em (838 ± 14)	Em‐t (730 ± 6)	↓	**<.001**
			Em‐l (794 ± 7)	Em‐t‐l (811 ± 8)	−	.365
	*E. microcarpa* (P)	Pollination	Et‐l (746 ± 13)	Em‐t‐l (811 ± 8)	↑	**.003**
Ovary wall thickness	*E. microcarpa* (P)	Oviposition	Et‐l (269 ± 15)	Em‐t‐l (311 ± 3)	↑	**<.001**
	*E. laeviclada* (C)	Oviposition	Em (306 ± 10)	Em‐l (342 ± 4)	↑	**<.001**
			Em‐t (316 ± 3)	Em‐t‐l (311 ± 3)	−	.173

Moreover, stylar pit depth decreased in response to the existence of *E. tertiaria* which exhibited pollination and oviposition via the stylar pit and increased in response to *E. microcarpa* pollinating via the stylar pit (Figure 9d; Table 6). This is interesting, since *E. tertiaria* larvae do also consume host seeds, and so we might expect *K. microcarpa* to evolve to reduce *E. tertiaria* oviposition as well. However, we still do not know whether the variation in stylar pit depth is a response to oviposition or pollination, since we lack of proboscis length data. Although both *E. microcarpa* and *E. tertiaria* performed pollinating behaviors, we did not estimate the pollination efficiency of each moth species. Locular oviposition requires cutting through the ovary wall, resulting in mechanical damage to the flowers, while stylar pit oviposition does not result in such damage. Consequently, we speculate that *E. tertiaria* may be the most beneficial pollinator, even though *E. microcarpa* occurred in almost all studied populations. Controlled experimental studies of the costs (e.g., seed consumption) and benefits (e.g., quality as pollinators) of these pollinators are needed. Our study provides new empirical evidence supporting the importance of community context for shaping flower traits, not only in pollination mutualism (Hollens‐Kuhr et al., 2021) but also in oviposition parasitism.

### 4.3 Mutualism and phenotypic differentiation

Ovipositor morphology is critical for host plant use and copulation, which may then affect plant–insect interactions and reproductive isolation (Althoff, 2014; Joy & Crespi, 2007). In the evolution of specialized yucca–yucca moth interactions, ovipositor length and morphology differentiated, suggesting adaptation to different oviposition behaviors, which may then lead to reproductive isolation and speciation (Althoff, 2014; Althoff et al., 2006). In this study, the pollinator *E. microcarpa* and cheater *E. laeviclada* exhibited shorter ovipositors and different oviposition behavior than the pollinator *E. tertiaria*, and the phallus length was correspondingly shorter in the former two species than that in *E. tertiaria* (Table 4), similar to the association between morphological differentiation and reproductive isolation in yucca moths (Althoff, 2014). However, it remains unclear how *E. microcarpa* and *E. laeviclada* (which have the same ovipositor length and locular oviposition in female moths, and the same phallus length in male moths) maintained reproductive isolation during the process of speciation and whether they have interspecific gene flow today. Either a period of allopatric isolation during the process of speciation or a host shift by one moth species from another species of *Kirganelia* to *K. microcarpa* seems likely; both have been demonstrated in other leafflower–leafflower moth systems (Hembry et al., 2013; Luo et al., 2017). Additionally, many genitalia traits such as serrature length, apophysis anterioris length, ova length, apophysis posterioris length in female moths and saccus length, costa length, tegumen length in male moths showed significant differences between these two species (Table S3), which might affect the mating process and reproductive isolation. Since these two species have the same oviposition niche, it seems likely that *E. microcarpa* and *E. laeviclada* compete strongly with each other (Hardin, 1960). If *E. microcarpa* is somehow the superior competitor, that might explain the low frequency of *E. laeviclada* in all populations expect Zhaoqing (Table 1), and contribute to maintaining the mutualism.

In this study, we did not estimate seed costs resulting from different *Epicephala* moth species, so we could not discuss to what extent the presence of the cheater will lead to mutualism breakdown. As our study focuses on one plant species and trait matching between multiple partners under different populations, future studies should focus on how and to what extent partner coexistence, especially of cheaters, affects mutualism maintenance, and variation in trait matching among different plant species in a phylogenetic context, especially since floral morphology and oviposition behavior vary greatly among leafflowers and leafflower moths. Furthermore, the population genetics of the three *Epicephala* species should be studied to estimated gene flow and reproductive isolation among species and populations. Our results show the potential for adaptation of moth ovipositor morphology to divergent oviposition behaviors and floral trait variation in response to different partners, which broaden our horizons on the coevolution between mutualistic partners and enhance our understanding of maintenance mechanisms in plant–animal mutualisms.

## AUTHOR CONTRIBUTIONS

**Kai Hao:** Data curation (supporting); formal analysis (lead); methodology (lead); visualization (lead); writing – original draft (lead); writing – review and editing (lead). **Ting‐Ting Liu:** Data curation (lead); investigation (lead); visualization (supporting). **David H. Hembry:** Conceptualization (equal); writing – original draft (supporting); writing – review and editing (supporting). **Shi‐Xiao Luo:** Conceptualization (equal); funding acquisition (lead); investigation (supporting); resources (lead); supervision (lead).

## FUNDING INFORMATION

This work was supported by the National Natural Science Foundation of China (No. 32270242, 31370268) to S.X.L.

## CONFLICT OF INTEREST STATEMENT

The authors declare that they have no conflict of interest.

## Supporting information


Appendix S1.

## Data Availability

No data are available.
