# Supplementary material for: Trait matching in a multi‐species geographic mosaic of leafflower plants, brood pollinators, and cheaters
Source: Ecol Evol. 2023 Jul 4;13(7):e10228. doi: 10.1002/ece3.10228 (PMC10318581; doi:10.1002/ece3.10228)
Supplement: Supplementary file 1 — Appendix S1. [file ECE3-13-e10228-s001.doc]

**
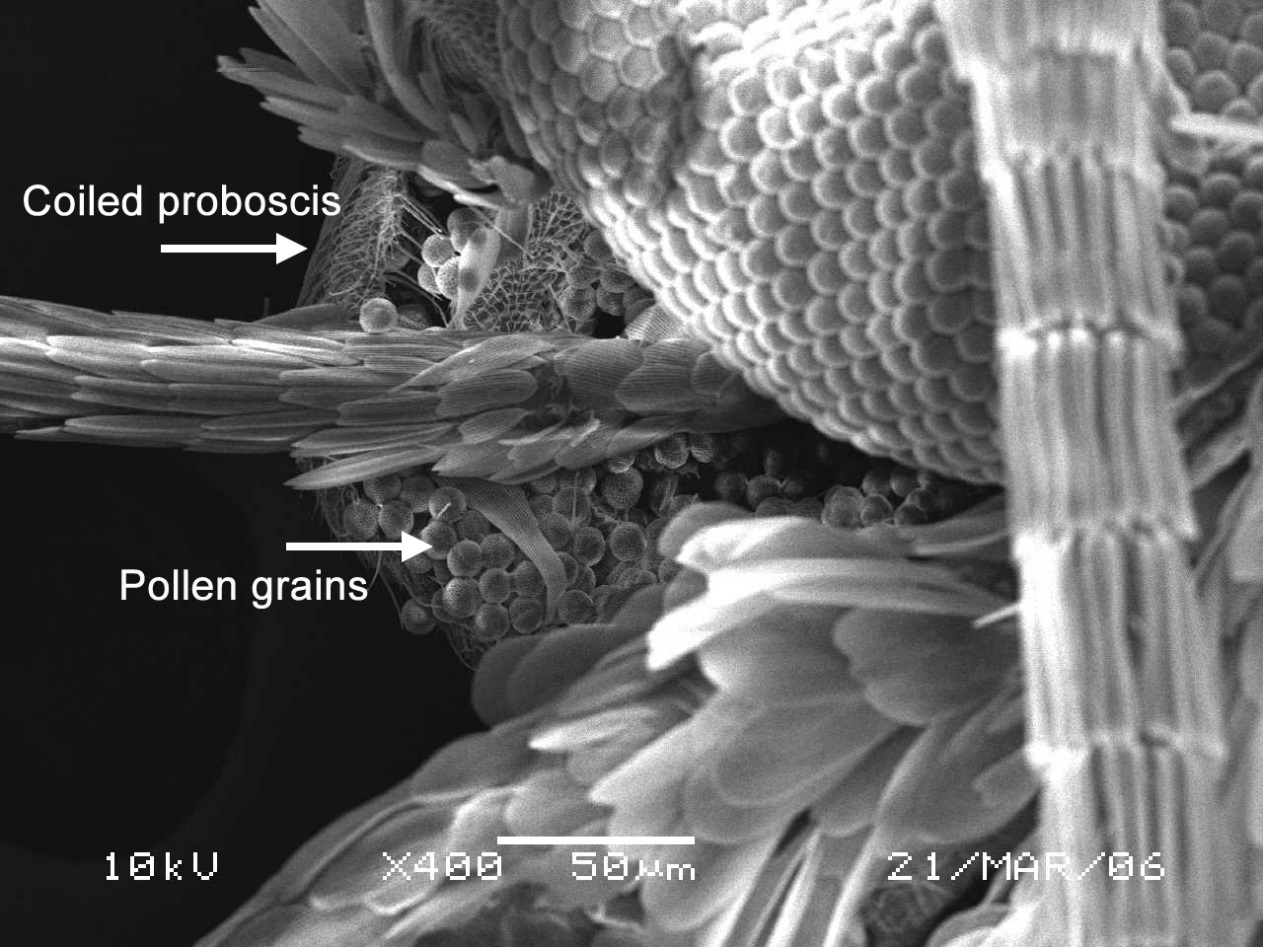
**

**Figure S1.** Pollen grains on the proboscis of a female *E. tertiaria* moth as seen with a scanning electron microscope (SEM).


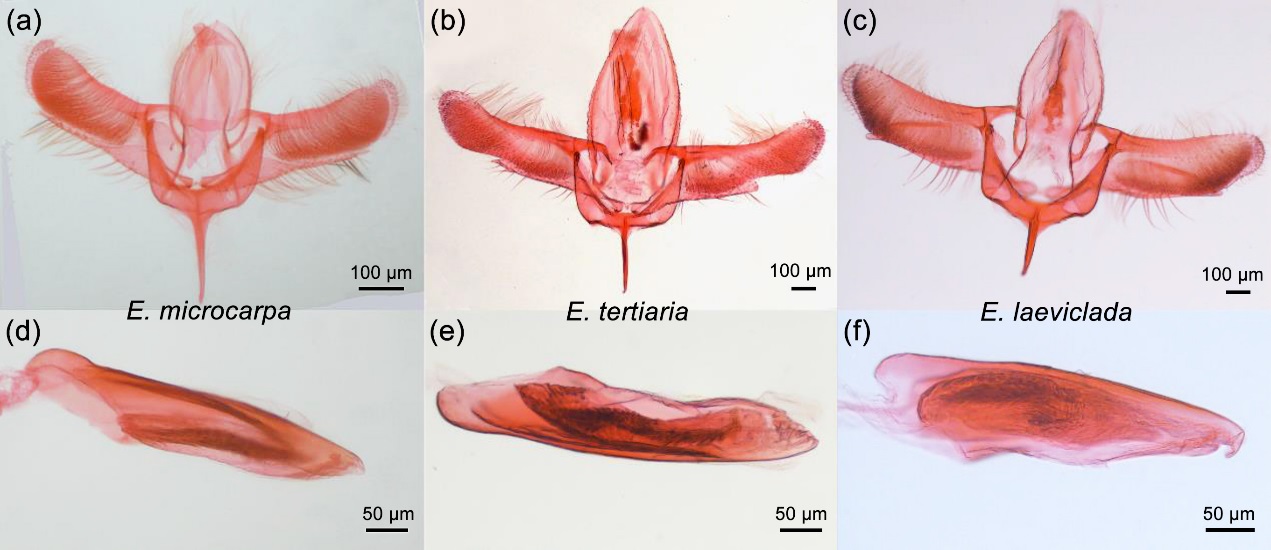


**Figure S2.** Morphology of valvae (a, b, c) and phallus (d, e, f) of male genitalia of *E. microcarpa*, *E. tertiaria* and *E. laeviclada*, respectively, stained with Eosin dye solution.


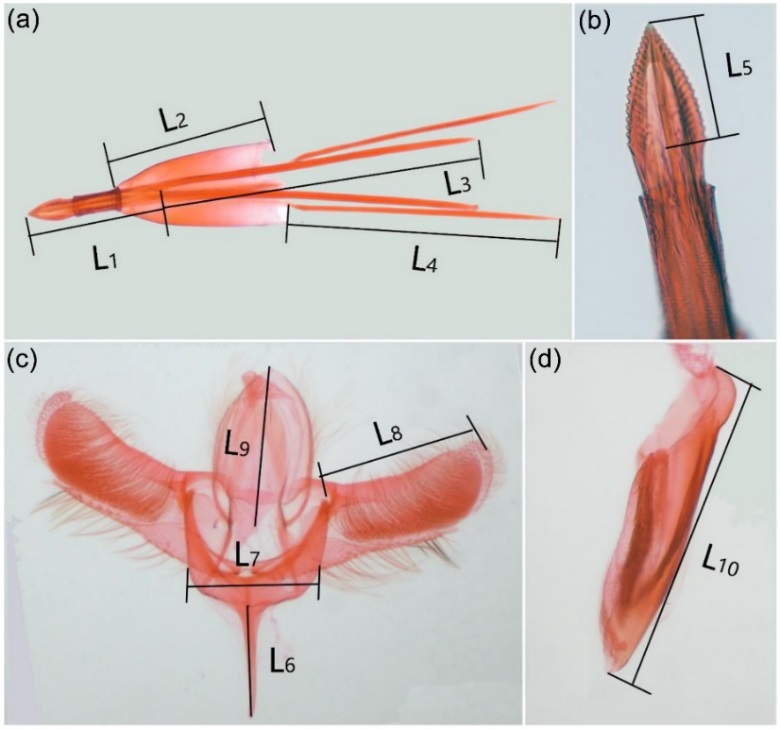


**Figure S3.** Schematic diagram showing the measurements of female genitalia traits (a: L1, ovipositor length; L2, lamella antevaginalis length; L3, apophysis anterioris length; L4: apophysis posterioris length; b: L5, keel length) and male genitalia traits (c: L6, saccus length; L7, vinculum width; L8, costa length; L9, tegument length; d: L10, phallus length) of *Epicephala* moths.


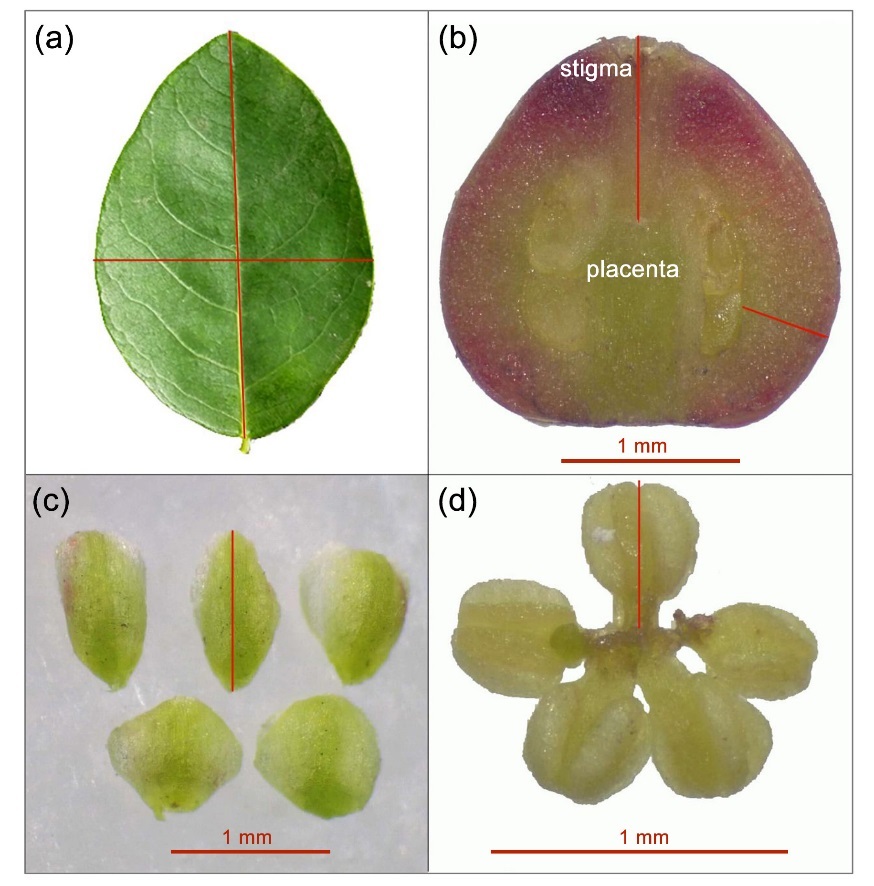


**Figure S4.** Schematic diagram showing the measurements of plant traits (a, leaf length and width; b, stylar pit depth (top) and ovary wall thickness (right); c, sepal length of male flower; d, stamen length) in *Kirganelia microcarpa*.

**Table S1.** Mean values (± SD) of floral traits and leaf traits in populations with different moth assemblages.

| Moth assemblages | Stylar pit depth (mm) | Ovary wall thickness (mm) | Stamen length  (mm) | Calyx length (mm) | Leaf length (cm) | Leaf  width  (cm) |
| --- | --- | --- | --- | --- | --- | --- |
| E_m_ | 0.84 ± 0.11 | 0.31 ± 0.07 | 0.51 ± 0.88 | 0.98 ± 0.17 | 3.38 ± 0.47 | 1.89 ± 0.23 |
| E_m-l_ | 0.79 ± 0.13 | 0.34 ± 0.07 | 0.42 ± 0.05 | 0.90 ± 0.14 | 4.41 ± 0.65 | 2.23 ± 0.35 |
| E_m-t_ | 0.73 ± 0.13 | 0.32 ± 0.08 | 0.50 ± 0.10 | 0.99 ± 0.31 | 4.49 ± 0.89 | 2.35 ± 0.41 |
| E_m-t-l_ | 0.81 ± 0.18 | 0.31 ± 0.07 | 0.44 ± 0.09 | 0.88 ± 0.28 | 4.57 ± 0.91 | 2.20 ± 0.43 |
| E_t-l_ | 0.75 ± 0.10 | 0.27 ± 0.12 | 0.45 ± 0.04 | 0.84 ± 0.08 | 4.65 ± 1.08 | 2.24 ± 0.27 |

**Table S2. Principal component analysis of floral traits and leaf traits among populations with different moth assemblages, in which stylar pit depth and ovary wall thickness were the dominant two traits.**

| Traits | PC 1 | PC 2 |
| --- | --- | --- |
| Stylar pit depth | 0.47617 | -0.15089 |
| ovary wall thickness | 0.48514 | -0.04595 |
| Stamen length | 0.34461 | 0.53051 |
| Calyx length | 0.46337 | 0.22708 |
| leaf length | -0.44503 | 0.30248 |
| leaf width | -0.0799 | 0.74204 |

**Table S3.** Comparisons of male and female genitalia traits (mean ± SE) among three *Epicephala* moth species under generalized linear model analyses. Bold values and different letters indicate significant differences at *P* < 0.05. The letters in brackets indicate moth pollinator (P) or cheater (C).

| **Genitalia traits (μm)** | ***Epicephala laeviclada* (C)** | ***E. microcarpa* (P)** | ***E. tertiaria* (P)** | **Wald χ^2^** | ***P*** |
| --- | --- | --- | --- | --- | --- |
| **Female moths** | n = 4 | n = 58 | n = 19 |  |  |
| Ovipositor length | 367.1^B^ ± 18.8 | 355.5^B^ ± 4.3 | 590.7^A^ ± 13.5 | 425.555 | **< 0.001** |
| Serrature length | 52.1^C^ ± 2.2 | 96.8^A^ ± 1.1 | 90.1^B^ ± 3.8 | 95.302 | **< 0.001** |
| Apophysis anterioris length | 671.2^C^ ± 19.5 | 846.0^B^ ± 8.0 | 907.3^A^ ± 17.9 | 51.578 | **< 0.001** |
| Ova length | 10384^C^ ± 29.1 | 1201.5^B^ ± 9.9 | 1498.0^A^ ± 18.8 | 222.757 | **< 0.001** |
| Apophysis posterioris length | 601.9^C^ ± 14.4 | 741.5^B^ ± 7.5 | 890.0^A^ ± 14.6 | 121.970 | **< 0.001** |
| Lamella antevaginalis length | 435.6^B^ ± 16.3 | 472.5^B^ ± 6.8 | 695.9^A^ ± 18.2 | 199.257 | **< 0.001** |
| Lap length (μm) | 1037.5^C^ ± 23.0 | 1213.9^B^ ± 12.3 | 1585.9^A^ ± 28.0 | 205.867 | **< 0.001** |
| **Male moths** | n = 8 | n = 54 | n = 17 |  |  |
| Phallus length | 406.5^B^ ± 11.2 | 405.0^B^ ± 3.8 | 444.8^A^ ± 8.2 | 20.874 | **< 0.001** |
| Saccus length | 144.9^B^ ± 9.2 | 167.8^A^ ± 2.9 | 155.8^B^ ± 5.4 | 11.368 | **0.003** |
| Costa length | 327.2^A^ ± 6.7 | 302.9^B^ ± 2.6 | 341.8^A^ ± 5.2 | 52.966 | **< 0.001** |
| Costa width | 116.7^A^ ± 2.8 | 120.5^A^ ± 1.8 | 94.9^B^ ± 3.0 | 56.843 | **< 0.001** |
| Tegumen length | 315.8^A^ ± 17.6 | 288.5^B^ ± 4.3 | 371.4^A^ ± 9.3 | 4.042 | 0.133 |
| Tegumen width | 177.1^A^ ± 9.1 | 161.0^A^ ± 3.3 | 186.2^A^ ± 6.4 | 2.296 | 0.317 |
| Vinculum width | 197.6^A^ ± 13.7 | 203.6^A^ ± 4.8 | 194.0^A^ ± 6.8 | 0.787 | 0.675 |
